# Ligand exchange engineering of FAPbI_3_ perovskite quantum dots for solar cells

**DOI:** 10.1007/s12200-022-00038-z

**Published:** 2022-09-23

**Authors:** Wentao Fan, Qiyuan Gao, Xinyi Mei, Donglin Jia, Jingxuan Chen, Junming Qiu, Qisen Zhou, Xiaoliang Zhang

**Affiliations:** School of Materials Science and Engineering, Beihang University, Beijing, 100191 China

**Keywords:** FAPbI_3_, Perovskite quantum dot, Antisolvent, Surface passivation, Solar cell

## Abstract

Formamidinium lead triiodide (FAPbI_3_) perovskite quantum dots (PQDs) show great advantages in photovoltaic applications due to their ideal bandgap energy, high stability and solution processability. The anti-solvent used for the post-treatment of FAPbI_3_ PQD solid films significantly affects the surface chemistry of the PQDs, and thus the vacancies caused by surface ligand removal inhibit the optoelectronic properties and stability of PQDs. Here, we study the effects of different anti-solvents with different polarities on FAPbI_3_ PQDs and select a series of organic molecules for surface passivation of PQDs. The results show that methyl acetate could effectively remove surface ligands from the PQD surface without destroying its crystal structure during the post-treatment. The benzamidine hydrochloride (PhFACl) applied as short ligands of PQDs during the post-treatment could fill the A-site and X-site vacancies of PQDs and thus improve the electronic coupling of PQDs. Finally, the PhFACl-based PQD solar cell (PQDSC) achieves a power conversion efficiency of 6.4%, compared to that of 4.63% for the conventional PQDSC. This work provides a reference for insights into the surface passivation of PQDs and the improvement in device performance of PQDSCs.

**Graphical abstract:**

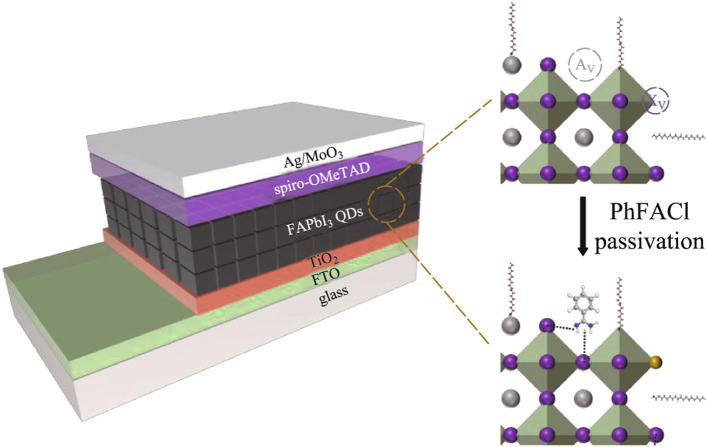

**Supplementary Information:**

The online version contains supplementary material available at 10.1007/s12200-022-00038-z.

## Introduction

Due to the combined advantages of perovskites and quantum dots (QDs), such as low cost, solution processability, tunable bandgap energy (*E*_g_) and high stability [1–9], perovskite QDs (PQDs) have received increasing attention for application in light-emitting diodes [10, 11], photodetector [12–14], and solar cells [15, 16]. Since the first PQD solar cells (PQDSCs) were successfully fabricated by Swarnkar et al. in 2016 [17], with the material synthesis improvement [18–20], post-treatment of PQDs [21–25], and tuning device structure of solar cells [26–29], the performance of inorganic CsPbI_3_ PQDSCs has considerably improved. Compared with CsPbI_3_ PQDs, formamidinium lead triiodide (FAPbI_3_) PQDs present a more ideal bandgap (~ 1.5 eV) for achieving highly efficient PQDSCs [30], and show superiority in phase stability [31], which is crucial for the future commercial application of solar cells. In addition, FAPbI_3_ PQDs show better stability compared with the bulk FAPbI_3_ perovskite due to the large contribution of surface energy and the presence of surface ligands [17, 31]. However, the power conversion efficiency (PCE) of FAPbI_3_ PQDSCs is generally lower than that of CsPbI_3_ PQDSCs, and is much lower than their theoretical efficiency.

In recent years, various approaches have been used to improve the photovoltaic performance and stability of solar cells containing FAPbI_3_ PQD light-absorbing layers. Xue et al. introduced a conjugated small-molecule into PQD solid films, which could act as a bridge for electron transport between PQDs. The FAPbI_3_ PQDSC with an efficiency of nearly 13% was then obtained [32]. Li et al. proposed a type of PQDSCs with an α-CsPbI_3_/FAPbI_3_ bilayer structure, which improved the carrier diffusion length and finally achieved the highest PCE of 15.6% [33]. Ji et al. fabricated a polymer-QDs bulk heterojunction hybrid layer at the interface between PQD solid film and hole transport layer (HTL), which significantly improved the short-circuit current density (*J*_sc_) and PCE of solar cells [34]. In addition, Ling et al. developed an approach to treat PQD solid films, i.e., a ligand exchange process followed by thermal annealing treatment (LE-TA), and showed that this approach can ultimately improve carrier transport in the PQD solid films [35]. However, we note that the hot-injection synthesis of FAPbI_3_ PQDs requires excessive oleic acid (OA) to ensure FA protonation and excessive A-site cations (molar ratio FA^+^/Pb^2+^  = 2.7) [36], which is significantly different from the case of CsPbI_3_ PQDs. Such intensive OA ligand anchored on the FAPbI_3_ PQD surface leads to significant differences in the surface chemistry between CsPbI_3_ and FAPbI_3_ PQDs [30]. So far the molecular design for surface passivation of FAPbI_3_ PQDs and the anti-solvent used during the post-treatment of FAPbI_3_ PQD solid film have rarely been studied.

Herein, we systematically investigated the anti-solvent used during the post-treatment of FAPbI_3_ PQD solid films and developed an effective surface passivation method for FAPbI_3_ PQDs. We selected the most suitable anti-solvent methyl acetate (MeOAc) from nearly 10 solvents with different polarities to make the best balance between the destroying the crystal structure and removing the surface ligand of FAPbI_3_ PQDs. We found that benzamidine hydrochloride (PhFACl) showed the best passivation effect on FAPbI_3_ PQDs, which can be attributed to the fact that formamidine group and Cl^−^ in PhFACl would simultaneously fill the A-site and X-site surface vacancies of FAPbI_3_ PQDs. Meanwhile, the aromatic unsaturated bonds in PhFACl could improve the electronic coupling of PQDs. Finally, with the good surface passivation of the PQDs, and with MeOAc used as anti-solvent and PhFACl acting as surface capping ligands, the optoelectronic properties of PQDs were substantially improved. Consequently, the PhFACl-based PQDSC achieved a high PCE of 6.4%, which was obviously elevated compared with that of the conventional FAPbI_3_ PQDSC (4.63%).

## Experimental section

### Materials synthesis

FAPbI_3_ PQDs were synthesized using the modified hot-injection method [36]. Formamidinium acetate (FAAc, 20 mmol), oleic acid (OA, 50.4 mmol) and 1-octadecene (ODE, 16 mL) were added together in a 100 mL 3-neck flask and degassed under vacuum at 50 ℃ for 1 h. This solution was heated to 130 ℃ under nitrogen and reacted for a while. Note that the FA-oleate precursor was heated to 120 ℃ under nitrogen before use.

PbI_2_ (0.94 mmol) and ODE (40 mL) were added to a 250 mL 3-neck flask and degassed under vacuum at 120 ℃ for 1 h. Then, OA and oleylamine (OAm) (OA/OAm = 2:1) were injected with constant nitrogen flow. When the solution was cooled to 80 ℃, the preheated FA-oleate precursor (5 mL) was rapidly injected. After a few seconds of the reaction, the mixture was cooled to room temperature using an ice-water bath.

2-pentanol (1:1 v:v ratio) was added to the PQD solution, and the mixture was centrifuged at 8000 r/min for 5 min. The separated PQDs were dispersed in hexane, re-precipitated with methyl acetate (MeOAc), and centrifuged at 8000 r/min for 2 min. The collected PQDs were dispersed in hexane. The resulting supernatant was finally stored at 4 ℃ for 24 h and centrifuged again before use.

### PQDSC fabrication

The etched Fluorine doped tin oxide (FTO) glass substrate was cleaned with sequential ultrasonication in detergent, deionized water, acetone and ethanol. To obtain a compact TiO_2_ layer acting as an electron transport layer (ETL) on the FTO substrate, a mildly acidic solution of titanium isopropoxide in ethanol was spin-coated on the FTO substrate at 2000 r/min for 30 s and then annealed at 500 ℃ for 1 h [37]. Before the deposition of PQD solid film, the compact TiO_2_ film was treated with ultraviolet-ozone for 20 min. The FAPbI_3_ PQDs were spin-coated on the top of the TiO_2_ layer at 2000 r/min for 40 s, and then an appropriate amount of anti-solvent was loaded onto the PQD film to remove the surface ligands. This process was repeated 3–5 times to build up a suitable thickness of a FAPbI_3_ PQD film and was performed under ambient conditions with a humidity of 15%–30%. The hole transport layer (HTL) was spin-coated on the PQD film at 3000 r/min for 30 s from a solution consisting of 72.3 mg of Spiro-OMeTAD, 30 μL of 4-tert-Butylpyridine (TBP), 17.5 μL of Li-TFSI (520 mg/mL in acetonitrile), 7.08 μL of FK209 solution (300 mg/mL in acetonitrile) and 1 mL of chlorobenzene. Finally, 10 nm MoO_3_ and 80 nm Ag electrode were evaporated onto the Spiro-OMeTAD layer.

### Measurements and characterization

The UV–visible absorption spectrum was tested using a UV–vis spectrophotometer (UV-3600, Shimadzu, Japan). The photoluminescence (PL) spectroscopy was measured using a spectrofluorometer with an excitation of 490 nm (LLS-490, Ocean Optical, USA). The morphology of PQDs was characterized using a transmission electron microscope (TEM) (JEM-2100, JEOL, Japan) at an accelerating voltage of 200 kV. The X-ray diffraction (XRD) pattern was measured with Rigaku D/max2500 using Cu Ka radiation (*λ* = 1.54178 Å) in an angle range of 5°–60°. The Fourier transform infrared (FTIR) spectrum was obtained using the Nicolet 6700 Fourier Transform Infrared Spectrometer in the transmittance mode. The scanning electron microscope (SEM) image was created using a scanning electron microscope (Zeiss SUPRA55) with an accelerating voltage of 10 kV. Under one sun illumination (AM1.5G, 100 mW/cm^2^) provided by a solar simulator (Enli Technology Co., Ltd. SS-F5-3A), the photocurrent density–voltage (*J–V*) curve of solar cells was measured using the Keithley 2400 digital source meter. The working area of the devices was 0.08 cm^2^, which was defined by a black metallic mask. The external quantum efficiency (EQE) spectrum was measured using an Enli Technology QE-R system. The transient photovoltage (TPV) and transient photocurrent (TPC) decay was measured using a compositive electrochemical workstation (Zahner Zennium CIMPS-pro).

## Results and discussion

**Fig. 1:**
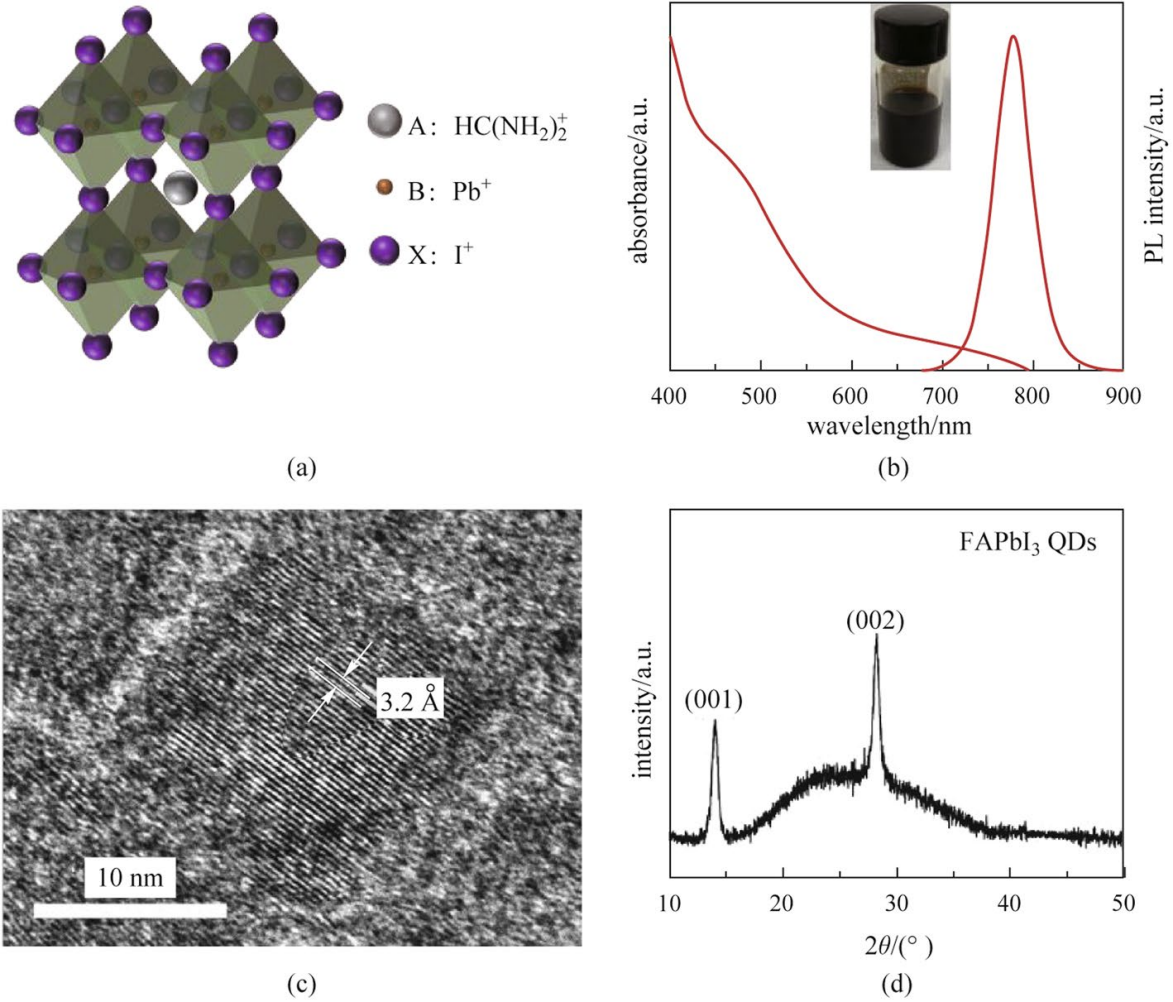
Fundamental properties of FAPbI_3_ PQDs. **a** Schematic diagram of the cubic phase of the FAPbI_3_ perovskite crystal structure. **b** UV–visible absorption and PL spectra of FAPbI_3_ PQDs. Inset shows the photograph of the PQD solution. **c** TEM image and **d** XRD pattern of FAPbI_3_ PQDs

FAPbI_3_ PQDs were synthesized using the above-mentioned modified hot-injection method, and the fundamental properties of the resulting PQDs were characterized. Figure 1a presents a schematic diagram of the cubic phase of the FAPbI_3_ perovskite crystal structure, showing the formamidine cation surrounded by the inorganic cages ([PbI_6_]^−^ octahedra) [38]. Compared with CsPbI_3_ perovskite, the Goldschmidt tolerance factor (GTF) of FAPbI_3_ perovskite was closer to 1 [39], demonstrating its better phase stability. Figure 1b shows the UV–visible absorption and PL spectra of FAPbI_3_ PQDs, and the inset shows the photograph of PQD solution. The PQDs exhibited a light absorption edge and PL emission peak at ~ 790 nm, and the solution color was brown-black. These results suggested that FAPbI_3_ PQDs were near-infrared luminescent materials that can absorb sunlight from visible and near-infrared wavelength regions [36, 40]. Figure 1c shows the TEM image of FAPbI_3_ PQDs, from which it can be seen that the PQDs were nearly cubic-shaped with a size of ~ 14 nm. In addition, the lattice parameter of FAPbI_3_ PQD was 3.2 Å, which is consistent with the literature [30]. Figure 1d shows the XRD pattern of FAPbI_3_ PQDs, which has obvious (001) and (002) diffraction peaks confirming that the as-synthesized FAPbI_3_ PQDs were cubic perovskite phase without other phases [41, 42]. The nanoscale size of PQDs endows the material with large surface tension, so that the PQDs show better stability than that of bulk perovskites and are less prone to phase transition [17].

**Fig. 2:**
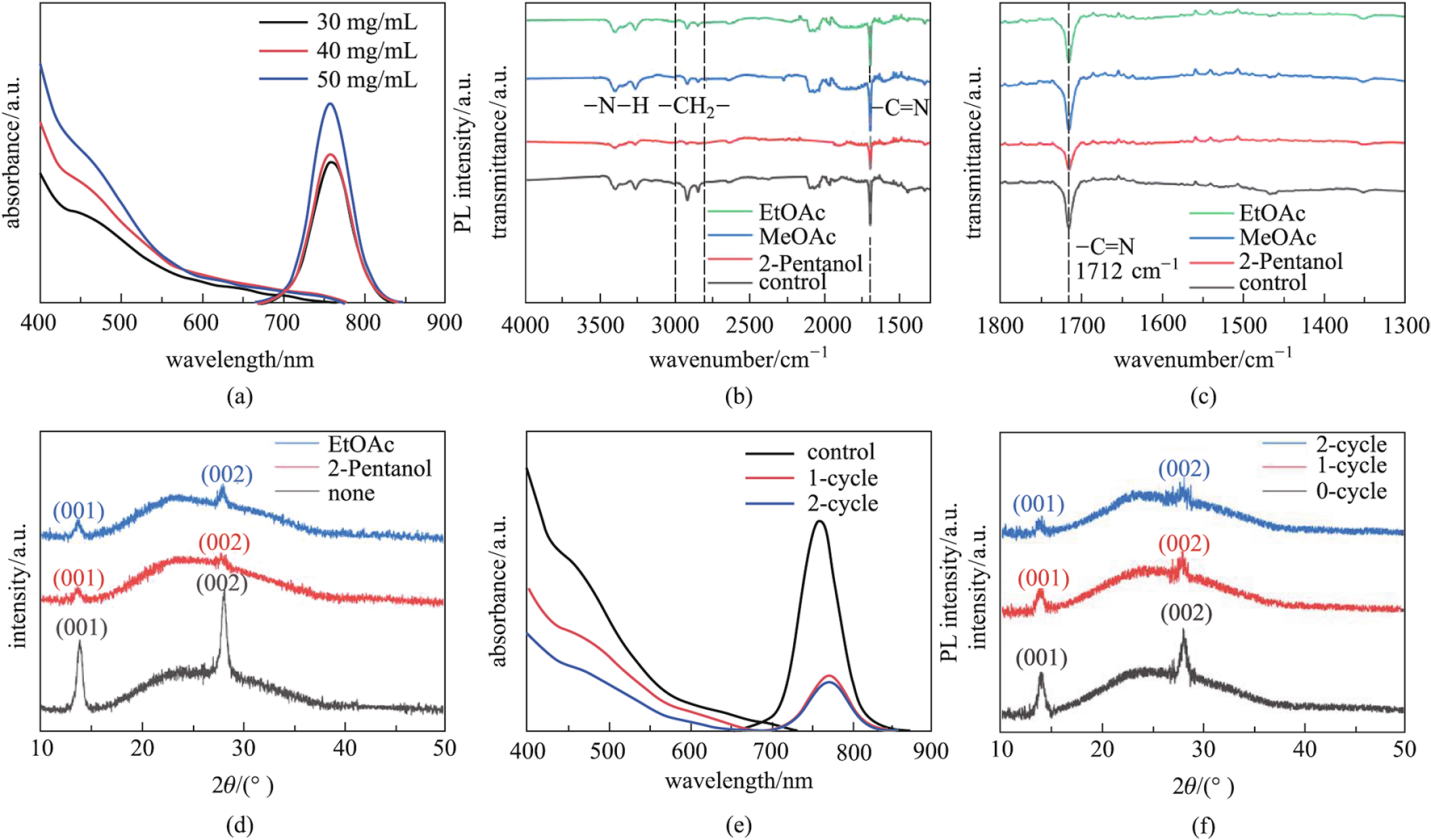
Surface ligand management for FAPbI_3_ PQDs. **a** UV–visible absorption and PL spectra of the FAPbI_3_ PQD solid film prepared with different concentrations of PQD solution. **b** FTIR spectra, **c** expanded view of FTIR spectra in the range of 1300 to 1800 cm^−1^ and **d** XRD pattern of the FAPbI_3_ PQD solid film treated with different anti-solvents. **e** UV–visible absorption and PL spectra, **f** XRD pattern of the FAPbI_3_ PQD solid film with increasing numbers of post-treatment cycles

Efficiently removing the long-chain insulating OA/OAm ligands from the surface of FAPbI_3_ PQDs without destroying the crystal structure of PQD is highly desirable, and thus we performed ligand removal of FAPbI_3_ PQDs using different anti-solvents. We first characterized the optical properties of the FAPbI_3_ PQD film prepared with the different concentrations of PQD solutions, and Fig. 2a shows the UV–visible absorption and PL spectra of these PQD solid films. It can be seen that with the increase of PQD concentration, the light absorption of these films gradually increased, but the basic characteristics of the UV–visible absorption spectra did not significantly change, suggesting that the FAPbI_3_ PQD solid films could remain in the cubic phase as the solution concentration changed. We selected various anti-solvents with different polarities to treat the PQD solid films and the photographs of these films after the treatment are shown in Additional file 1: Fig. S1. The UV–visible absorption and PL spectra of the FAPbI_3_ PQD solid film treated with different anti-solvents are presented in Additional file 1: Figs. S2 and S3, respectively, and the relative polarities of these anti-solvents are summarized in Additional file 1: Table S1. From these results, we found that 2-pentanol, MeOAc, and ethyl acetate (EtOAc) show potential to be used as anti-solvents for the post-treatment of PQD solid films. We then paid further attention to these anti-solvents when studying the PQD solid films and related solar cells.

The ability of an anti-solvent to remove surface ligands and its effect on the structure of FAPbI_3_ PQDs are of great importance for the construction of PQD solid film with high conductivity and stability. The FTIR spectra of the FAPbI_3_ PQD solid film treated with 2-pentanol, MeOAc, and EtOAc are shown in Fig. 2b. Figure 2c shows the enlarged FTIR spectra in the wavenumber range of 1300–1800 cm^−1^. For comparison, the spectra of PQD solid film without the anti-solvent treatment is also included in the figures and named as the control sample. The vibration band at 1712 cm^−1^ belongs to the − C = N group, which is derived from the FA^+^ in PQDs [30]. The signal did not change significantly after the treatment with MeOAc and EtOAc. However, after 2-pentanol treatment, the signal intensity was significantly weakened, which may be due to the high polarity of 2-pentanol that removed the FA^+^ from the surface of FAPbI_3_ PQDs. The signal of the vibration band between 2800 and 3000 cm^−1^ (belonging to −CH_2_− group from OA and OAm) was significantly decreased after these anti-solvent treatments [25, 30]. Notably, after the 2-pentanol treatment, the vibration band of −CH_2_− decreased most obviously among these samples, indicating that 2-pentanol could more effectively remove OAm ligands from the surface of FAPbI_3_ PQDs. We note that 2-pentanol has different functional groups compared with MeOAc and EtOAc, which may be the cause of its efficient removal of OAm ligands. Based on the above results, it seems that the OAm ligands on the surface of FAPbI_3_ PQDs are difficult to remove, which is not conducive to improving the PCE of FAPbI_3_ PQDSCs [43]. The XRD pattern of the FAPbI_3_ PQD solid film with different anti-solvent treatments is shown in Fig. 2d. After the treatment with EtOAc and 2-pentanol, the intensity of the (001) and (002) diffraction peaks of PQDs decreased and no impurity peaks were observed, indicating that these anti-solvents did not induce the phase transition of the PQDs. Therefore, MeOAc may be more suitable as an anti-solvent for the treatment of the FAPbI_3_ PQD solid film.

Subsequently, the washing times of the anti-solvent were explored to further optimize the post-treatment of the FAPbI_3_ PQD solid film. Figure 2e shows the UV–visible absorption and PL spectra of the FAPbI_3_ PQD solid film with increasing number of post-treatment cycles. The result reveals that the light absorption of the FAPbI_3_ PQD solid film gradually deteriorated with the increase of washing times, which may be because the anti-solvent dissolved a small amount of FAPbI_3_ PQDs. Compared with the 1-cycle washing, the PL intensity of the film with 2-cycle washing did not change significantly, indicating that repeated washing had a limited effect on removing the surface ligands of FAPbI_3_ PQDs. The XRD pattern of the FAPbI_3_ PQD solid film with different anti-solvent washing times is shown in Fig. 2f, from which it can be seen that the second washing had less effect on the crystalline of FAPbI_3_ PQDs than the first one. These results demonstrate that the surface ligand removal of FAPbI_3_ PQDs may be related to the polarity or functional groups rather than the number of washing times of the anti-solvent.

**Fig. 3:**
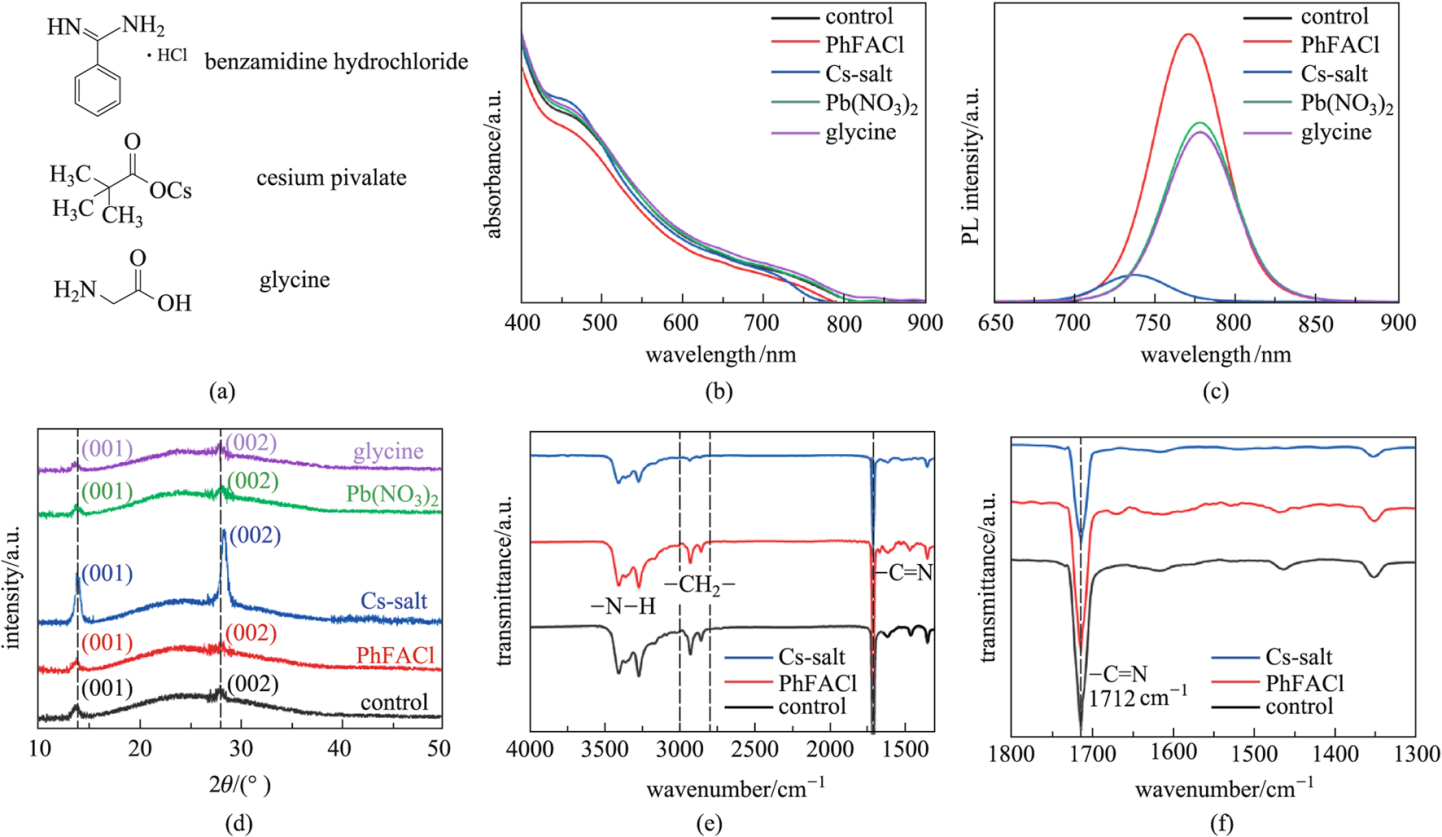
Ligand exchange of FAPbI_3_ PQDs. **a** Chemical structures of the molecules used for ligand exchange of PQDs. **b** UV–visible absorption, **c** PL spectra, **d** XRD pattern, **e** FTIR spectra, **f** zoomed-in view, in the range of 1300 to 1800 cm^−1^, of FTIR spectra of the FAPbI_3_ PQD solid films with different molecule ligands on the PQD surface

A large number of organic insulating ligands are located on the surface of FAPbI_3_ PQDs. These insulating ligands can improve the stability of PQDs (compared to bulk perovskites), whereas they can hinder the charge carrier transport between PQDs. As such, for solar cell applications, these insulating ligands need to be substituted with short ones to facilitate charge carrier transport within the PQD solid films. Previous work has found that, after removing the insulating ligands with anti-solvent, a large number of A-site and X-site defects appear on the surface of PQDs, and these defects become nonradiative recombination centers of photogenerated charge carriers [16]. Therefore, to replace the organic insulating ligands on the surface of PQDs without significantly deteriorating the optoelectronic properties of PQDs, we selected several molecules for ligand exchange of PQDs. The chemical structures of these molecules are shown in Fig. 3a. The formamidine group in PhFACl has the same structure as the A-site cation of FAPbI_3_ perovskite. Chloride ions (Cl^−^) and the X-site anion of FAPbI_3_ perovskite are both halogen ions, which could stabilize the perovskite crystals of PQDs [44, 45]. The Cesium pivalate (named as Cs-salt) contains Cs^+^ and carboxyl (RCOO^−^), and the former could effectively fill the A-site defects of PQDs [46], while the latter is often used to fill the X-site defects due to the functional group of oleate [47, 48]. According to literature reports [49], glycine can be used as dual-passivation ligands to passivate the surface defects of CsPbI_3_ PQDs, and we explored whether it could effectively passivate FAPbI_3_ PQDs. In addition, the traditional saturated MeOAc solution of Pb(NO_3_)_2_ and anhydrous MeOAc-based FAPbI_3_ PQD solid films were also used in the comparison of the passivation effect of these molecules.

The effect of surface passivation of the above-mentioned small molecules on the optical properties of FAPbI_3_ PQD solid films was investigated. The UV–visible absorption and PL spectra of the FAPbI_3_ PQD solid films treated with MeOAc solutions containing different molecule ligands are shown in Fig. 3b and c, respectively. The PL intensity of Glycine-based PQD solid film was very close to that of the Pb(NO_3_)_2_-based PQD solid film, and the purple (Glycine) and black (Control) lines in Fig. 3c almost coincided. The light absorption edge and PL emission peak of the PhFACl-based PQD solid film were slightly blue-shifted compared with that of the control sample. This was due to the interaction between the benzamidine in PhFACl and the corner-sharing [PbI_6_]^4−^ octahedron [50, 51], as well as the fact that Cl^−^ could fill the surface X-site defects of PQDs and exchange into the perovskite interior [52, 53]. Moreover, the PhFACl-based PQD solid film had the highest PL intensity in the PL spectra, indicating that PhFACl could effectively fill the surface defects of FAPbI_3_ PQDs, thereby increasing the radiative recombination of PQD solid films, which is crucial for improving charge carrier transport of solar cell devices [54]. In addition, the light absorption edge and PL emission peak of the Cs-salt-based PQD solid film were blue-shifted compared with that of the control sample, which may be due to a small amount of Cs^+^ entering FAPbI_3_ PQDs, forming organic–inorganic hybrid (FA_1−*x*_Cs_*x*_PbI_3_) PQDs and thus increasing their the bandgap energy [39, 53]. Notably, the Cs-salt-based PQD solid film had the lowest intensity in the PL spectra, indicating that it may introduce more defects while replacing the original OA/OAm ligands. Meanwhile, we found that the exchange reaction of FA^+^ and Cs^+^ in PQDs was very violent, which may provide a potential avenue for the synthesis of FA_1−*x*_Cs_*x*_PbI_3_ PQDs. The UV–visible absorption and PL spectra of glycine and Pb(NO_3_)_2_-based PQD solid films were not significantly different from the control samples, which suggested that the surface chemistries of FAPbI_3_ PQDs and CsPbI_3_ PQDs are quite different and surface passivation of FAPbI_3_ PQDs may require a new molecular design.

On the other hand, these small molecules may damage the perovskite structure of FAPbI_3_ PQDs. Thus the XRD patterns of FAPbI_3_ PQD solid films with different post-treatment were measured, as shown in Fig. 3d. It can be seen that after the Cs-salt treatment, the diffraction peaks of (001) and (002) were shifted to higher angles in the XRD patterns and the intensity was significantly increased, which confirmed the previous supposition that Cs^+^ penetrated the FAPbI_3_ PQDs forming organic–inorganic hybrid (FA_1−*x*_Cs_*x*_PbI_3_) PQDs. It also demonstrated that the dispersed PQDs may be agglomerated after Cs-salt treatment. Other samples did not show significant differences and all corresponded to the cubic phase perovskite. The FTIR spectra of these PQD solid films were also characterized and the results are shown in Fig. 3e and f. Specifically, the peaks at ~ 1560 cm^−1^, attributing to the conjugated aromatic ring in PhFACl [19], was not observed for PhFACl-based PQD solid film. This may be due to the high signal intensity of the vibration band at 1712 cm^−1^ (belonging to −C=N group, which is from FA^+^ in FAPbI_3_ PQDs), masking the weaker peaks. In contrast to the control sample, the signal intensity of the vibration band between 2800 and 3000 cm^−1^ in Cs-salt-based PQD solid film dropped significantly, indicating that the OA and OAm ligands on the surface of FAPbI_3_ PQDs were largely removed. The signal of the vibration band at 1712 cm^−1^ was significantly decreased in Cs-salt-based PQDs, indicating decreased content of FA^+^ on the surface of the Cs-salt-based PQDs. The vibration band between 3300 and 3500 cm^−1^ could be ascribed to −N−H group [30, 31], and the weakening of its signal indicated the reduction of OAm ligands or FA^+^ on the surface of PQDs. The results of FTIR spectra were consistent with the previous analysis that Cs^+^ in the Cs-salt partially replaced FA^+^ on the surface of the PQDs. This resulted in the reduction of OA ligands, since the A-site cation on the surface of PQDs was in the form of OA-A (eg, oleic acid-formamidine) [30]. OAm ligands occupied the A-site vacancies on the surface of PQDs with protonated oleylamine group [19], and the filling of A-site defects by Cs^+^ also removed OAm ligands from the PQD surface.

**Fig. 4:**
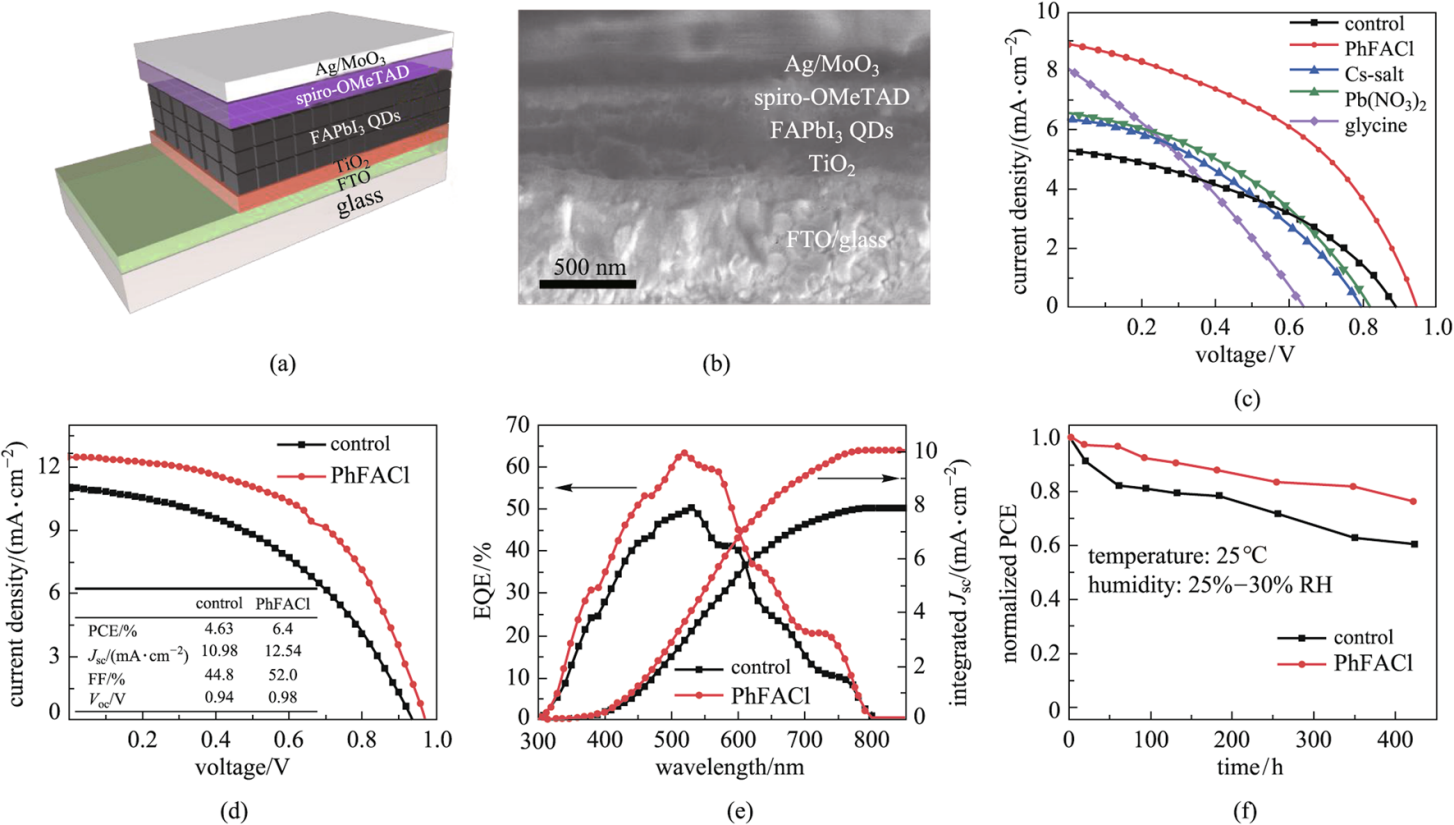
Device architecture and photovoltaic performance of PQDSCs. **a** Device architecture and **b** cross-sectional SEM image of the PQDSC. **c** *J–V* curves of PQDSCs with different post-treatment processes. **d** *J–V* curves and inset summarized the photovoltaic parameters of PQDSCs, **e** EQE spectra and integrated *J*_sc_ curves, and **f** stability of control and PhFACl-based PQDSCs

To investigate the effect of surface passivation of PQDs on the performance of solar cell devices, PQDSC with a planar architecture of Glass/FTO/TiO_2_/PQDs/Spiro-OMeTAD/MoO_3_/Ag was fabricated as shown in Fig. 4a. The TiO_2_ layer was used as the ETL, and the Spiro-OMeTAD layer was applied as the HTL in the device. The corresponding cross-sectional SEM image of the device is shown in Fig. 4b. Figure 4c presents the *J–V* curves of PQDSC, which were recorded under the AM1.5G illumination with a light intensity of 100 mW/cm^2^. Initially, the thickness of the light-absorbing layer and the environmental conditions for device fabrication were not precisely controlled, resulting in generally low PCE of FAPbI_3_ PQDSCs. The PhFACl-based PQDSC shows the best photovoltaic performance among these devices. After the optimization, PhFACl-based PQDSC with a high PCE of 6.40% was obtained (Fig. 4d). Compared with the control device, the improved PCE in PhFACl-based PQDSC was mainly due to the improvement of *J*_sc_ and FF, which may result from the improved electronic coupling of PQDs. However, we found that the PhFACl-based PQDSC showed hysteresis-like behaviour in the *J–V* curves as voltage scanning from different directions, and the causes are still unknown so far (Additional file 1: Fig. S4).

The EQE spectra and integrated *J*_sc_ curves of the control and PhFACl-based PQDSCs are shown in Fig. 4e. The EQE spectra of the PhFACl-based PQDSCs exhibit apparent improvements across the entire photo-response region compared with the control device, indicating that the charge collection efficiency was improved [55]. Figure 4f shows the evolution of the normalized PCE of un-encapsulated devices with storage under ambient conditions (20%–30% RH, ~ 25 °C). It can be seen that ~ 60% of the initial PCE was retained in the control PQDSCs, whereas the PhFACl-based PQDSC maintains ~ 76% of the initial PCE after aging for 400 h. Therefore, the PhFACl can improve the photovoltaic performance of PQDSCs by filling the A-site and X-site defects on the surface of PQDs, which diminishes charge carrier recombination, and meanwhile the benzamidine can act as the surface ligand to protect the PQDs, improving the stability of PQDSCs.

**Fig. 5:**
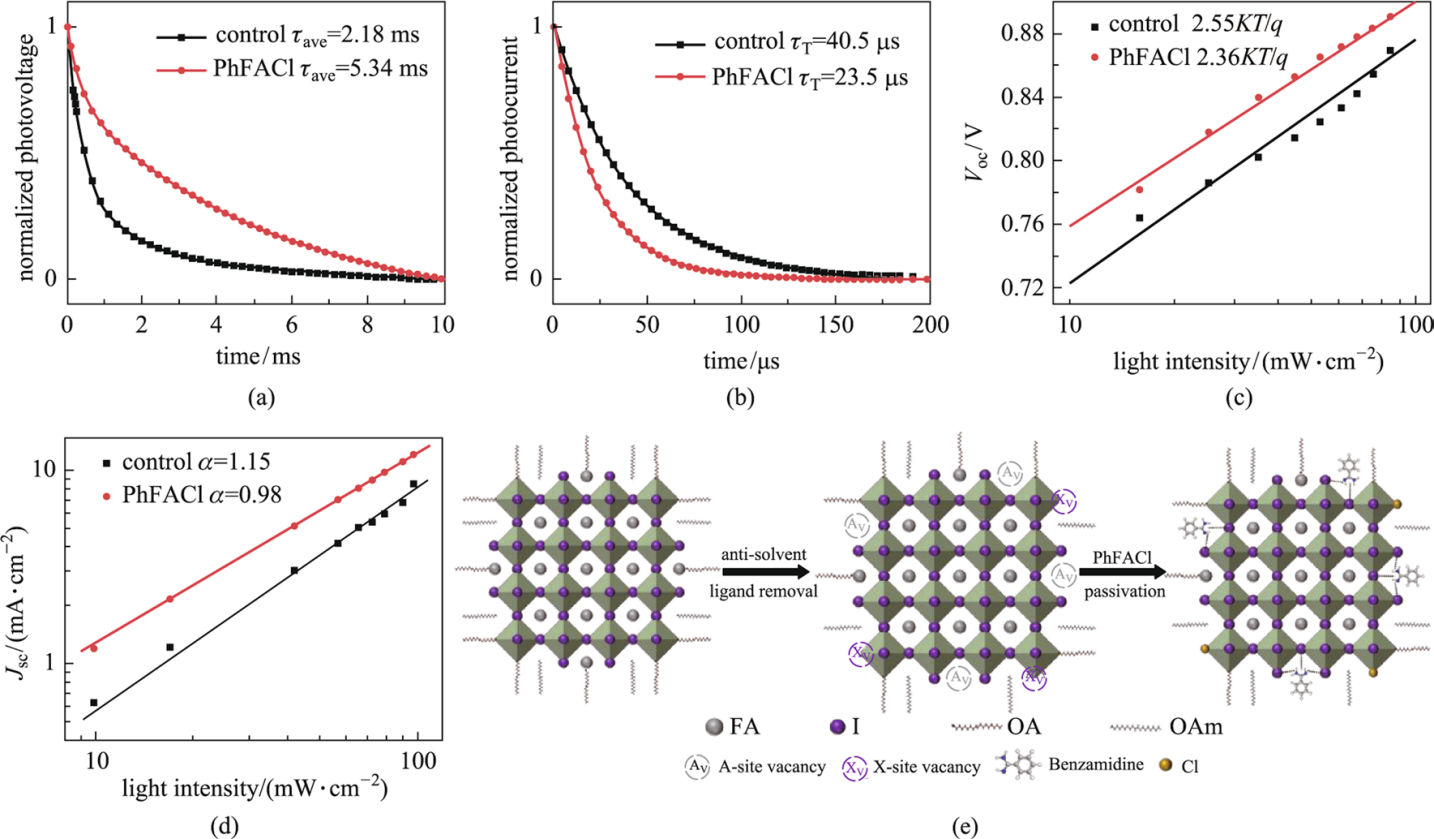
Charge carrier dynamics of PQDSCs. **a** TPV and **b** TPC curves of control and PhFACl-based PQDSCs. **c** *V*_oc_ and **d** *J*_sc_ of control and PhFACl-based PQDSCs as a function of incident light intensity. **e** Schematic diagram of the passivation of the PQD surfaces using PhFACl

The above findings suggest that improved surface passivation of PQDs with PhFACl can enhance the photovoltaic performance and stability of the device, which may be due to the diminished charge carrier recombination. To confirm such a hypothesis, the charge carrier dynamic of PQDSCs was analyzed. Figure 5a shows the TPV curves of the control and PhFACl-based PQDSCs, and bi-exponential functions were used to fit the curves. The fitting parameters are detailed in Additional file 1: Table S2. The results show that the PhFACl-based PQDSC possessed a longer charge carrier lifetime (5.34 ms) than that of the control PQDSC (2.18 ms), which was consistent with PL results of PQD solid films. These results indicate that the charge carrier recombination of PQDSCs was significantly reduced [15]. Similar to TPV, TPC is usually used to study charge carrier dynamics in solar cells. The TPC curves of the control and PhFACl-based PQDSCs were tested, as shown in Fig. 5b, and the exponential functions and fitting parameters are summarized in Additional file 1: Table S3. The results reveal that the TPC decay of the PhFACl-based PQDSC (23.5 μs) was significantly shorter than that of the control PQDSC (40.5 μs), which indicated that the transport of photogenerated charge carriers was significantly improved within the PhFACl-based PQDSC [25]. This was because benzamidine has a conjugated aromatic ring, and the existence of unsaturated bonds can effectively promote the electronic coupling between individual PQDs and thus improve the transport of photogenerated charge carriers.

To further study the charge carrier dynamics of FAPbI_3_ PQDSCs, the open-circuit voltage (*V*_oc_) and *J*_sc_ as a function of the light intensity were measured. The results of light intensity-dependent *V*_oc_ can be fitted using the following equation,

1 $$\begin{array}{c}{V}_{\mathrm{oc}}=\frac{nKT}{q}\mathrm{ln}\phi +C,\end{array}$$

where *n* is the diode ideality factor, *K* is the Boltzmann constant, *T* is the temperature in Kelvin, *q* is the elementary charge, and *ϕ* is the light intensity. A lower diode ideality factor usually represents less trap-assisted nonradiative recombination in solar cells [56]. Figure 5c shows the *V*_oc_ as a function of the light intensity for control and PhFACl-based PQDSCs. The values of the calculated slope are 2.55*KT/q* and 2.36*KT/q* for control and PhFACl-based PQDSCs, respectively, which indicates that PhFACl can effectively reduce trap-assisted nonradiative recombination in FAPbI_3_ PQDSCs.

The light intensity-dependent Jsc is processed using the following equation,

2 $$\begin{array}{c}{J}_{\mathrm{sc}}{\propto \phi }^{\alpha },\end{array}$$

where *α* is the exponent and *ϕ* is the light intensity. If the exponent is close to 1, the relationship between photogenerated charge carrier recombination and collection in the solar cell is less affected by the light intensity [57]. As shown in Fig. 5d, after PhFACl passivation, the exponent of PQDSCs changed from 1.15 to 0.98, which was very closer to 1, indicating that the charge carrier recombination of the PhFACl-based PQDSCs was significantly suppressed. The dark *J*–*V* curves of the control and PhFACl-based PQDSCs are shown in Additional file 1: Fig. S5, also suggesting fewer defects in the PhFACl-based PQDSCs [49].

Based on the above studies, Fig. 5e shows the schematic diagram of surface passivation of the PQDs using PhFACl as a ligand. When the PQD solid film was washed with anti-solvent, part of the surface ligands was removed, whereas a large number of surface defects (including A-site and X-site) were simultaneously introduced. The formamidine group in PhFACl has a similar structure as the FA^+^ at the A-site in the PQDs, which could interact with the corner-sharing [PbI_6_]^4−^ octahedron, occupying the A-site vacancy on the surface of the PQD. Meanwhile, the Cl^−^ can also fill the X-site defects on the surface of the PQD. Therefore, defect-induced nonradiative recombination is substantially diminished within the PhFACl-based PQD solid film. In addition, the unsaturated bonds in the aromatic ring can also act as a bridge for carrier transport and improve the electronic coupling between FAPbI_3_ PQD, facilitating charge carrier transport within the PhFACl-based PQD solid film and thus improving the photovoltaic performance of PQDSCs.

## Conclusions

In summary, the solvent engineering of FAPbI_3_ PQDs with different molecular passivation was studied for solar cell applications. Screening from nearly 10 anti-solvents, MeOAc showed promising as an anti-solvent for the post-treatment of PQDs without deteriorating the crystal structure of PQDs. After passivating the PQD surface with PhFACl as short ligands in combination with MeOAc applied as anti-solvent, the optoelectronic properties of PQD solid films were largely improved with substantially diminished trap-assisted recombination. As a consequence, the PhFACl-based PQDSC yielded a PCE of 6.40%, which was a significant improvement compared with that of 4.63% for the control device. The charge carrier dynamic of PQDSCs was studied in detail, which revealed that the improved performance in PhFACl-based PQDSC was attributed to improved charge carrier transport, resulting from improved surface passivation of PQDs using PhFACl as passivators. We believe that this work will offer a deep understanding of the surface passivation of PQDs for solar cells and other optoelectronic devices.

## Supplementary Information

Below is the link to the electronic supplementary material.

Additional file 1: **Fig. S1. **Photograph of the FAPbI_3_ PQD solid film with different anti-solvent treatments. The solid films in the 1st line are prepared with 1 layer of PQDs, and those in the 2nd are 2 layers of PQDs. **Fig. S2. **UV-visible absorption spectra of the FAPbI_3_ PQD solid films with different anti-solvent treatments. **Fig. S3. **PL spectra of the FAPbI_3_ PQD solid films with different anti-solvent treatments. **Fig. S4. ***J*–*V* curves of conventional and PhFACl-based PQDSCs under reverse and forward voltage scanning directions. **Fig. S5. **Dark *J–V *curves of conventional and PhFACl-based PQDSCs. **Table S1**. Summary of anti-solvent relative polarity. The relative polarity of mixed solvent was determined from the arithmetic average of the solvent volume ratios. **Table S2**. Fitting parameters of the TPV curves of conventional and PhFACl-based PQDSCs. **Table S3**. Fitting parameters of the TPC curves of conventional and PhFACl-based PQDSCs.
