# Supplementary material for: Ligand exchange engineering of FAPbI3 perovskite quantum dots for solar cells
Source: Front Optoelectron. 2022 Sep 23;15(1):39. doi: 10.1007/s12200-022-00038-z (PMC9756204; doi:10.1007/s12200-022-00038-z)
Supplement: Supplementary file 1 — Additional file 1: Fig. S1. Photograph of the FAPbI3 PQD solid film with different anti-solvent treatments. The solid films in the 1st line are prepared with 1 layer of PQDs, and those in the 2nd are 2 layers of PQDs. Fig. S2. UV-visible absorption spectra of the FAPbI3 PQD solid films with different anti-solvent treatments. Fig. S3. PL spectra of the FAPbI3 PQD solid films with different anti-solvent treatments. Fig. S4. J–V curves of conventional and PhFACl-based PQDSCs under reverse and forward voltage scanning directions. Fig. S5. Dark J–V curves of conventional and PhFACl-based PQDSCs. Table S1. Summary of anti-solvent relative polarity. The relative polarity of mixed solvent was determined from the arithmetic average of the solvent volume ratios. Table S2. Fitting parameters of the TPV curves of conventional and PhFACl-based PQDSCs. Table S3. Fitting parameters of the TPC curves of conventional and PhFACl-based PQDSCs. [file 12200_2022_38_MOESM1_ESM.pdf]

Supplementary Information

Ligand Exchange Engineering of $FAPbI_3$ Perovskite Quantum Dots for Solar Cells

Wentao Fan, Qiyuan Gao, Xinyi Mei, Donglin Jia, Jingxuan Chen, Junming Qiu, Qisen Zhou, and Xiaoliang Zhang*

School of Materials Science and Engineering, Beihang University, Beijing 100191, China

Email: Xiaoliang.zhang@buaa.edu.cn

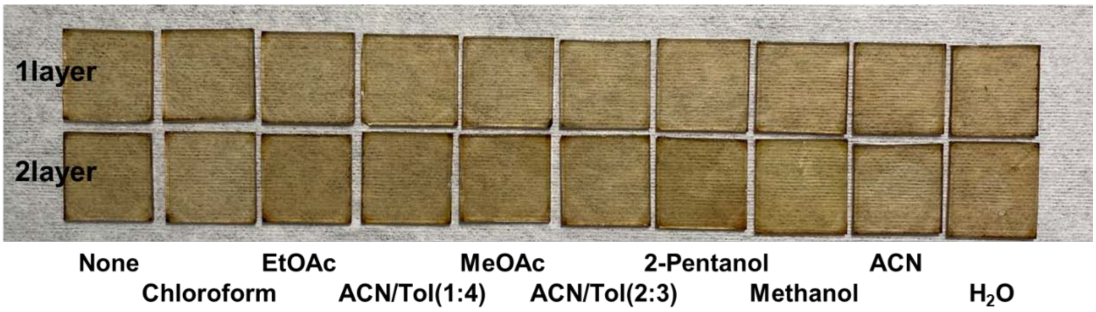

Fig. S1 Photograph of the $FAPbI_3$ PQD solid film with different anti-solvent treatments. The solid films in the 1st line are prepared with 1 layer of PQDs, and those in the 2nd are 2 layers of PQDs.

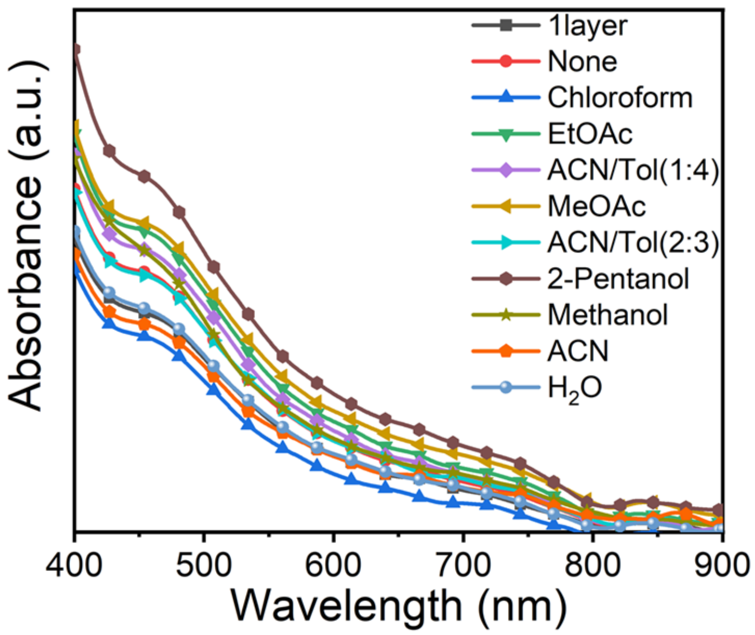

Fig. S2 UV-visible absorption spectra of the $FAPbI_3$ PQD solid films with different anti-solvent treatments.

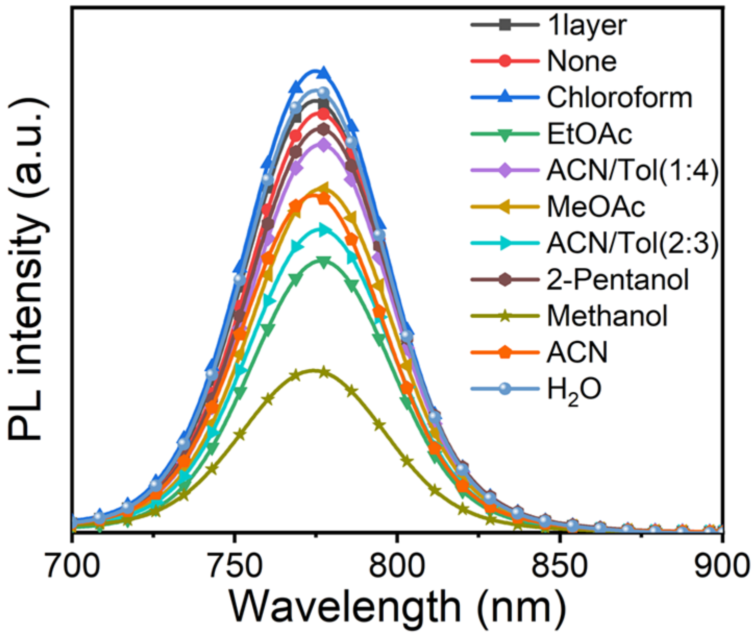

Fig. S3 PL spectra of the $FAPbI_3$ PQD solid films with different anti-solvent treatments.

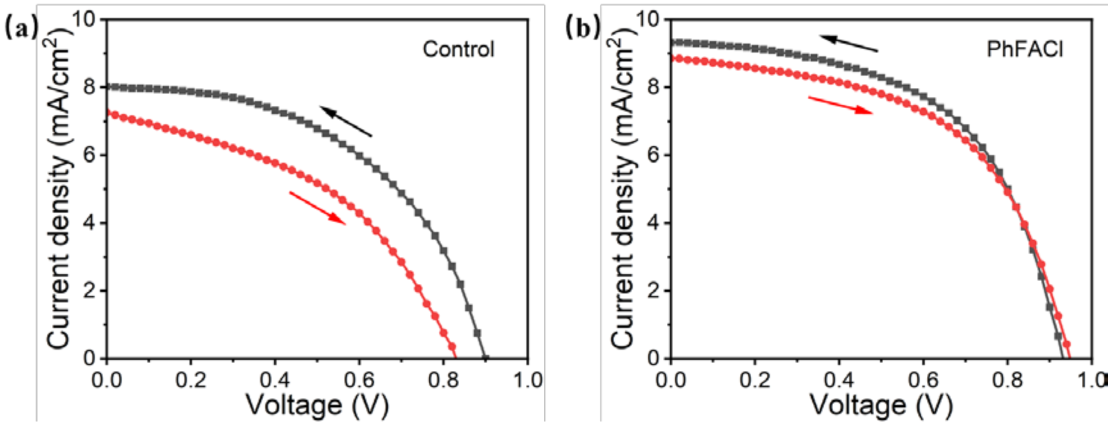

Fig. S4 *J*-*V* curves of conventional and PhFACl-based PQDSCs under reverse and forward voltage scanning directions.

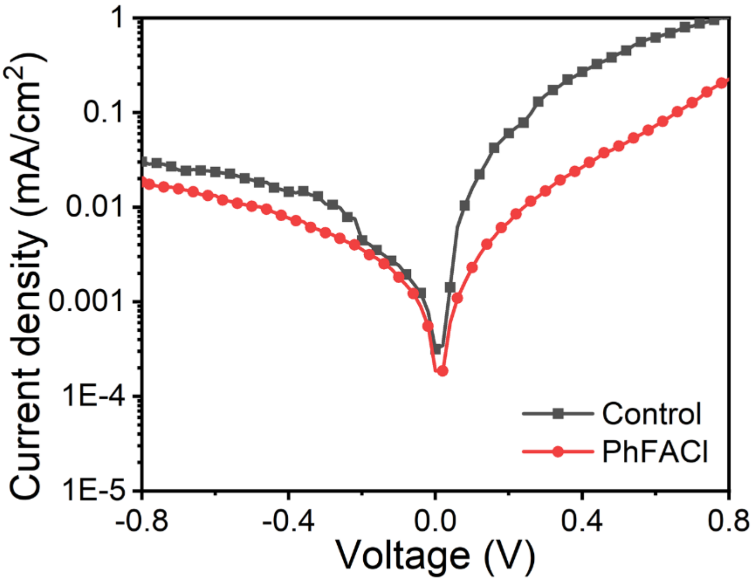

Fig. S5 Dark *J*-*V* curves of conventional and PhFACl-based PQDSCs.

Table S1. Summary of anti-solvent relative polarity. The relative polarity of mixed solvent was determined from the arithmetic average of the solvent volume ratios.[1]

**Name**	**Relative polarity**	**Name**	**Relative polarity**
ACN/toluene (v:v 1:4)	0.171	EtOAc	0.228
ACN/toluene (v:v 2:3)	0.243	Chloroform	0.259
MeOAc	0.287	ACN	0.46
2-pentanol	0.488	Methanol	0.762
H_2O	1		

Table S2. Fitting parameters of the TPV curves of conventional and PhFACl-based PQDSCs.

Parameter	Control	PhFACl
**A**	0.49	0.17
**τ_1(ms)**	0.44	0.34
**B**	0.22	0.53
**τ_2(ms)**	2.80	5.43
**C**	0.04	0.21
**τ_{ave}(ms)**	2.18	5.34

The data of the TPV measurement is fitted using the following equation:

$$V_{oc} = A\,exp\left(-\frac{t}{\tau_1}\right) + B\,exp\left(-\frac{t}{\tau_2}\right) + C$$

where t is the decay time, τ_1 and τ_2 are the decay lifetimes, and A, B, and C are constants.

The average lifetime, τ_{ave}, is calculated using the following equation:

$$\tau_{ave} = \frac{A{\tau_1}^2 + B\tau_2^2}{A\tau_1 + B\tau_2}$$

Table S3. Fitting parameters of the TPC curves of conventional and PhFACl-based PQDSCs.

Parameter	Control	PhFACl
**A**	0.22	0.35
**τ_T(ms)**	40.5	23.5
**B**	0.005	0.006

The data of the TPC measurement is fitted using the following equation:

$$J_{sc} = A\ exp\left(-\frac{t}{\tau_T}\right) + B$$

where t is the decay time, τ_T is the decay lifetime, and A and B are constants.

References

1. Xue, J., Lee, J.-W., Dai, Z., Wang, R., Nuryyeva, S., Liao, M.E., Chang, S.-Y., Meng, L., Meng, D., Sun, P., Lin, O., Goorsky, M.S., Yang, Y.: Surface Ligand Management for Stable FAPbI3 Perovskite Quantum Dot Solar Cells. Joule. 2, 1866-78 (2018). http://dx.doi.org/10.1016/j.joule.2018.07.018
